# Quantifying hexagonal stacking in diamond

**DOI:** 10.1038/s41598-019-46556-3

**Published:** 2019-07-17

**Authors:** Mara Murri, Rachael L. Smith, Kit McColl, Martin Hart, Matteo Alvaro, Adrian P. Jones, Péter Németh, Christoph G. Salzmann, Furio Corà, Maria C. Domeneghetti, Fabrizio Nestola, Nikolay V. Sobolev, Sergey A. Vishnevsky, Alla M. Logvinova, Paul F. McMillan

**Affiliations:** 1 Department of Earth and Environmental Sciences, University of Pavia, Via A. Ferrata 1, 27100 Pavia, Italy; 2 Department of Chemistry, University College London, 20 Gordon Street, London, WC1H 0AJ UK; 3 Department of Earth Sciences, University College London, 5 Gower Place, London, WC1E 6BS UK; 4 Institute of Materials and Environmental Chemistry, Research Centre for Natural Sciences-HAS, Magyar tudósok körútja 2, 1117 Budapest, Hungary; 5 Department of Geosciences, University of Padova, Via G. Gradenigo 6, 35131 Padova, Italy; 6 V.S. Sobolev Institute of Geology and Mineralogy, Siberian Branch of Russian Academy of Sciences, Koptyug Ave. 3, Novosibirsk, 90630090 Russia; 7 Novosibirsk State University, str. Pirogova 2, Novosibirsk, 630090 Russia

**Keywords:** Mineralogy, Mineralogy

## Abstract

Diamond is a material of immense technological importance and an ancient signifier for wealth and societal status. In geology, diamond forms as part of the deep carbon cycle and typically displays a highly ordered cubic crystal structure. Impact diamonds, however, often exhibit structural disorder in the form of complex combinations of cubic and hexagonal stacking motifs. The structural characterization of such diamonds remains a challenge. Here, impact diamonds from the Popigai crater were characterized with a range of techniques. Using the MCDIFFaX approach for analysing X-ray diffraction data, hexagonality indices up to 40% were found. The effects of increasing amounts of hexagonal stacking on the Raman spectra of diamond were investigated computationally and found to be in excellent agreement with trends in the experimental spectra. Electron microscopy revealed nanoscale twinning within the cubic diamond structure. Our analyses lead us to propose a systematic protocol for assigning specific hexagonality attributes to the mineral designated as lonsdaleite among natural and synthetic samples.

## Introduction

Impact cratering is one of the most common geological processes resulting in accretion and remodelling of planetary surfaces, and contributing to the development of their atmosphere and even biological evolution. Assessing the frequency of impacts on a tectonically inactive body, such as the Moon, is relatively straightforward through inspection of the planetary surface. However, on Earth, impact structures are typically obscured or have been obliterated by tectonic activity and volcanic resurfacing, erosion and burial processes, and surface vegetation. In order to deduce the presence of a terrestrial impact site it is often necessary to be guided by markers present in the mineral record that provide evidence of shock metamorphism within the country rock or among remnants derived from the impactor^1^. When a sufficiently large projectile (e.g., >50 m for stony and >20 m for iron meteorites) passes through the atmosphere without significant deceleration or disruption, it impacts the surface at velocities typically >11 km s^−1^. This causes shock waves that radiate into the target at velocities on the order of several km s^−1^ producing pressures (*P*), temperatures (*T*) and strain fields that are orders of magnitude greater than those achieved by endogenic metamorphism. The extreme *P*-*T* conditions generated can cause melting and even vaporization of refractory phases, and result in structural and phase transformations among minerals of the impacted rocks. One such mineralogical marker is the presence of features indicating hexagonal symmetry in the X-ray diffraction patterns of diamonds recovered from the impact site^2,3^. Such features have been associated with the presence of lonsdaleite, a metastable form of diamond that has also been reported to form in static as well as dynamic shock high-pressure high-temperature (H*P*H*T*) laboratory experiments^4–13^.

Lonsdaleite-bearing diamonds have been found in samples collected from large impact craters such as Popigai in Siberia^14–19^, in addition to the type of mineral phase described from Canyon Diablo (Barringer Meteor Crater, Arizona)^3,4,20,21^. The cubic structure of diamond is based on tetrahedrally-bonded carbon atoms linked into a 4-connected network, with all C-C bonds and C-C-C angles identical at 1.54 Å and 109.47°, respectively. The structure is usefully described in terms of layers of corrugated six-membered ring units with a cyclohexane “chair” conformation that are stacked with identical orientation perpendicular to the cubic (111) axis, accompanied by a shift half-way across the diagonal of the six-membered rings^22^. This constitutes the thermodynamically stable form of carbon over a very wide range of pressures and temperatures^23^. An alternative motif is achieved by successively stacking mirror images of the layers on top of each other resulting in hexagonal symmetry when propagated in three dimensions along the stacking direction. The two structures correspond to 3C and 2H motifs, respectively, using Ramsdell symbols to designate the layered stacking polytypes.

A form of diamond exhibiting hexagonal features in its X-ray diffraction pattern was described by Bundy and Kasper for a phase produced by H*P*H*T* treatment of graphite^4^. The powder X-ray diffraction data could be associated with a hexagonal phase having unit cell parameters *a* = *b* = 2.51 Å and *c* = 4.12 Å, and a density similar to that of cubic diamond (3.51 g cm^−3^). An analogous material was also reported from shock compression studies^24^. Diamonds containing similar hexagonal X-ray signatures were then found in samples from the Canyon Diablo and Goalpara (Assam, India) meteorite impact sites^3^. The hexagonal diamond form was associated with the 2H stacking polytype of elemental carbon by analogy with wurtzite *vs* sphalerite structures of tetrahedrally bonded compounds such as SiC and ZnS. It was assigned the mineral name lonsdaleite in recognition of the contributions of Kathleen Lonsdale to crystallography^2^. Other layered arrangements are also found among SiC and other tetrahedrally bonded materials, with different repeat sequences of the cubic (*c*) and hexagonal (*h*) stacking motifs occurring along the stacking direction giving rise to 4H, 6H, 8H and other polytypes. These have been reported to occur among diamond materials prepared by vapor deposition techniques^25–27^.

Because of its importance as a mineralogical marker for shock impact events as well as possibly leading to a useful class of technological materials, it is essential to fully understand and describe the nature of the lonsdaleite structure, how it is formed and how it can be controlled. Questions have arisen concerning the identification of the mineral lonsdaleite with a purely hexagonally crystalline structure^11,22,28^. In addition, the interpretation of lonsdaleite as evidence for shock metamorphism has been questioned recently^29^. The presence of hexagonal features in natural impact diamonds is typically interpreted as the result of shock transformation from graphite present in country rocks, although shock compression of diamond and graphite present within the impactor is also considered^2,4,30–33^. In most samples, the hexagonal phase has been reported to occur in close association with cubic diamond and graphite, often as inclusions within the shocked crystals. Although monocrystalline lonsdaleite was reported to occur^34^, the electron diffraction data used as evidence can also be interpreted as twinned cubic diamond following the description presented by Németh *et al*.^11^. In a transmission electron microscopy (TEM) study of materials recovered from a static compression experiment, Shiell *et al*. claimed to have produced nearly pure (90%) hexagonal diamond^35^. However, the diffraction data exhibited only amorphous rings that were more consistent with the signatures of quenchable amorphous diamond as reported by Zeng *et al*.^36^. Kraus *et al*.^12^ likewise claimed the synthesis of pure hexagonal diamond by shock compression of a pyrolytic graphite sample. However, that description was based on the observation of a doubled feature in the X-ray diffraction pattern that could equally well be interpreted as two peaks of coexisting cubic diamond structures formed within different strain regimes that can occur during shock processes. Although Turneaure *et al*.^13^ reported the finding of pure hexagonal diamond from highly oriented pyrolytic graphite, the orientational relationship deduced from the X-ray reflections contradict the direct TEM observations of the graphite to diamond transition reported by Garvie *et al*.^33^. Furthermore, the reflections attributed to hexagonal diamond are in fact consistent with nanotwinned cubic diamond reported by Németh *et al*.^11^.

Németh *et al*. conducted a detailed analysis of high resolution transmission electron microscopy (HRTEM) images and diffraction data to show that the characteristic features of lonsdaleite are best interpreted in terms of the cubic diamond structure containing multiple stacking faults and twinning defects, rather than as an sp^3^-bonded layered phase with hexagonal symmetry^11,28^. Salzmann *et al*.^22^ and later Jones *et al*.^16^ applied the MCDIFFaX technique^37^ to analyse the X-ray diffraction data of natural and laboratory-produced diamonds in order to determine their average hexagonality *Φ*_h_, i.e. the fraction of hexagonal stacking, and differentiate between ordered *vs* disordered stacking sequences. This approach allows fitting of the diffuse scattering features that arise from stacking disorder. The MCDIFFaX results led to the construction of a “stackogram” with poles extending along a line containing a random sequence of stacking between the fully cubic and fully hexagonal end-members, with two other extrema extending towards a physical mixture of locally ordered domains (i.e., (*ccc*)_n_ coexisting with (*hh*)_n_) *vs* a fully ordered polytype structure (*chch*)_n_^16,22^. The results were reported along lines of constant *Φ*_h_ in terms of *Φ*_cc_ (i.e., the probability of cubic stacking occurring after a previous cubic event) and *Φ*_hc_ (i.e., the probability of cubic stacking consecutive to a previous hexagonal event). The hexagonality *Φ*_h_ is calculated from the switching probabilities according to *Φ*_h_ = *Φ*_ch_/(*Φ*_hc_ + *Φ*_ch_). Those analyses of the X-ray diffraction data revealed that the impact diamond samples contain multiple intermediate stacking sequences that occupy a range of average hexagonality indices with a distribution of *Φ*_cc_ *vs Φ*_hc_ values. It was concluded that lonsdaleite should best be described as stacking disordered diamond.

Microbeam Raman spectroscopy has also been used to detect the presence of hexagonal lonsdaleite *vs* cubic diamond structures in natural and laboratory samples^38–40^. The 3C diamond phase exhibits a single Raman peak (T_2g_ symmetry) at 1332 cm^−1^. Subtle shifts in the peak position along with peak broadening can reveal the presence of non-isotropic strains, associated with impurities or structural defects within the samples. However, characteristic broadening and development of asymmetry in the main Raman band extending to lower wavenumbers is also used to diagnose the appearance of “lonsdaleite”-like structures. *Ab initio* calculations predict that the 2H structure with *P*6_3_/*mmc* space group and *Z* = 4 atoms in its primitive unit cell should exhibit three Raman peaks with A_1g_, E_1g_ and E_2g_ symmetry at the Brillouin zone centre^16,41–44^. Deconvolution of the Raman spectra of natural diamonds produced by shock events, including samples from the Popigai impact crater, have been used to deduce the presence of coexisting 3C and 2H diamonds^40,45^. However, this interpretation does not agree with the MCDIFFaX analysis of the X-ray data for similar suites of samples that indicate the presence of complex interlaced hexagonal/cubic stacking regimes^16,22^, or with HRTEM data indicating that the hexagonal diffraction features arise from nanoscale twinning and layer stacking defects within the cubic 3C diamond structure^11,28^.

**Figure 1:**
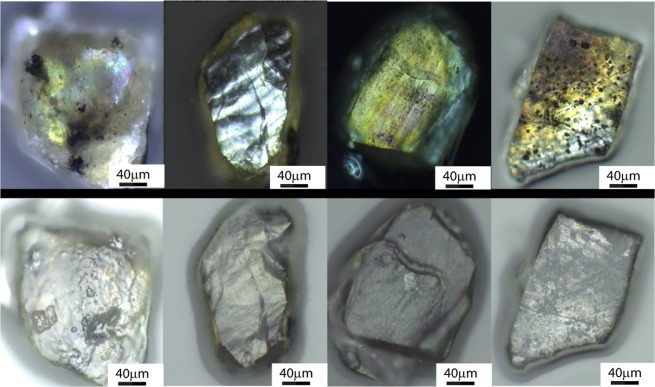
Optical images of representative Popigai impact diamonds examined in this study obtained using (top) transmitted and (bottom) reflected light. Striations in the images correspond to train bands and occasional black spots to graphite contained within the samples.

Here we present a systematic analysis of a suite of diamond samples (Fig. 1) from the Popigai impact crater using a combination of X-ray diffraction with MCDIFFaX analysis, followed by Raman spectroscopy accompanied by a series of density functional theory (DFT) calculations for different hexagonal/cubic stacking sequences, and HRTEM studies of the shocked phases. Our results allow us to suggest a new systematic protocol to be used to describe hexagonal diamond and lonsdaleite structures found in nature or created in the laboratory.

## Results

### Hexagonality indices from X-ray diffraction analysis

**Figure 2:**
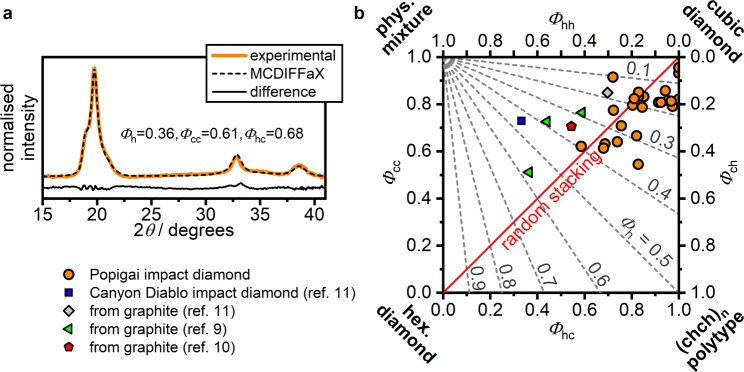
Characteristics of cubic-hexagonal stacking disorder in Popigai diamonds. (**a**) X-ray diffraction pattern of a Popigai diamond sample fitted using MCDIFFaX^37^. (**b**) First-order stacking probabilities of all Popigai samples shown on a ‘stackogram’^37^. Lines of constant hexagonality are shown as grey dashed lines whereas the solid red line indicates random stacking.

We obtained powder X-ray diffraction patterns of 23 Popigai impact diamond samples and analysed the data using the MCDIFFaX approach. A typical diffraction pattern displaying the diffuse diffraction features arising from the stacking disorder is shown in Fig. 2a.

The results from the fits allowed us to plot their *Φ*_h_, *Φ*_cc_ and *Φ*_hc_ values on a stackogram, where they are compared with previous data obtained from Canyon Diablo and laboratory-shocked samples (Fig. 2b). As mentioned previously, the leading diagonal extends between the cubic 3C and hexagonal 2H diamond structures. The other two poles indicate the clustering of cubic *vs* hexagonal stacking sequences, ranging between a physical mixture of extended (*ccc*)_n_ and (*hh*)_n_ stacking sequences to the fully ordered (*chch*)_n_ polytype. The hexagonality of a sample, *Φ*_h_, can be read off from their position with respect to the dashed lines of constant *Φ*_h_. The data for the Popigai samples all lie near the top right corner with *Φ*_h_ ranging from almost zero to 0.4 and a large number of samples located close to *Φ*_h_ = 0.2. These are distinct from the Canyon Diablo sample^22^ with *Φ*_h_ ~ 0.45 which is located towards the physical mixture pole of the stackogram. This observation is certainly related to the fact that the formation of the two impact craters involved very different shock conditions^15,46^.

Our results indicate that using MCDIFFaX to analyse X-ray diffraction data and to plot the findings on a stackogram is a very powerful method to obtain detailed insights into the overall hexagonality of a diamond sample as well as the degree of clustering of the hexagonal *vs* cubic layer sequences. In addition to this observation, Figure S1 demonstrates a useful relationship between the crystallographic *c*/*a* ratio and the hexagonality of a diamond sample.

### Effect of stacking disorder on the Raman spectra

**Figure 3:**
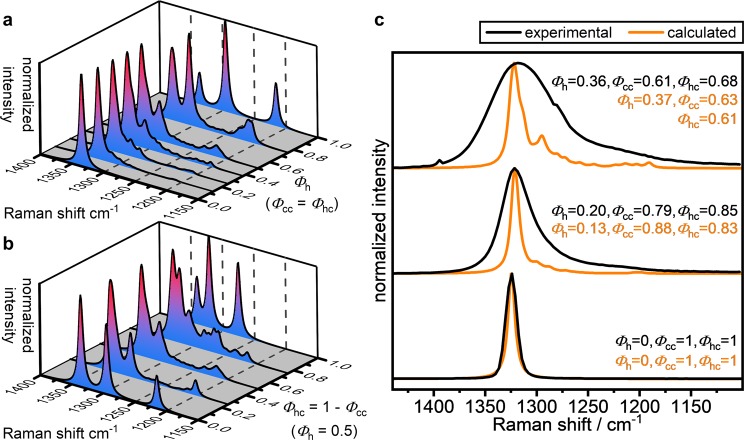
Effect of stacking disorder on the Raman spectra of diamond exhibiting cubic-hexagonal features in their stacking pattern. (**a**) Calculated Raman spectra along the random stacking line and (**b**) along the line of constant 50% hexagonality. (**c**) Comparison of experimental Raman spectra of Popigai and cubic diamonds with corresponding calculated spectra of similar hexagonalities. The calculated spectra in part (**c**) have been downshifted by 10 cm^−1^ to allow direct comparison with the experimentally measured Raman shifts.

Further information can be gained from the interpretation of the Raman spectra of diamond samples produced during shock events^40,45^. However, here we advise caution in applying simple interpretations based on physical mixtures of 3C/2H diamonds to the observed spectra. Because our X-ray data analysis showed that the Popigai samples studied contained a range of nanoscale layer stacking structures, we carried out DFT calculations to predict the Raman signatures of various 48-layer model structures with ordered *vs* disordered stacking regimes corresponding to specific positions on the stackogram along the random stacking line (*Φ*_hc_ = *Φ*_cc_) and the 50% isohexagonality line (*Φ*_h_ = 0.5). Simulated Raman spectra from these model structures are presented in Fig. 3a (*Φ*_hc_ = *Φ*_cc_) and 3b (*Φ*_h_ = 0.5). Moving along the random stacking line starting from the 3C polytype, as an increasing fraction of hexagonal stacking is introduced to the models, the spectra develop Raman intensity in the ~1290–1305 cm^−1^ region, which ultimately becomes the strongest feature for *Φ*_h_ >0.75. Weaker features emerge at 1210 cm^−1^, that also grow in intensity as *Φ*_h_ increases. The full range of experimental and calculated spectra is presented in the Supporting Information.

Considering structures along the 50% isohexagonality line, starting at *Φ*_hc_ = 0, the spectrum of the physical mixture exhibits three peaks and clearly demonstrates the overlap of the T_1g_ cubic diamond peak with the E_1g_ peak of the 2H polytype, which gives rise to a single feature at ~1335 cm^−1^. As *Φ*_hc_ increases, this band decreases in intensity and a new peak at ~1320 cm^−1^ emerges on the shoulder of the main peak at *Φ*_hc_ >0.75 which ultimately becomes the most intense feature for the (*chch*)_n_ polytype. The peak at ~1210 cm^−1^, present in the physical mixture, decreases in intensity along the *Φ*_h_ = 0.5 line as *Φ*_hc_ increases and is not present in the (*chch*)_n_ polytype. A comparison of the peak intensity in the ~1210 cm^−1^ region against the ~1270 cm^−1^ region may therefore give information on the local domains present for samples with a given degree of hexagonality. Intensity at ~1210 cm^−1^ can be considered diagnostic of (*hh*) stacking elements, whereas intensity at ~1270 cm^−1^ indicates ordered (*ch*) stacking. Stacking disorder generally manifests itself by weak Raman features at around 1250 cm^−1^, whereas zero intensity is observed in this region for all the ordered polytypes.

Experimental Raman spectra of two Popigai and a cubic diamond are shown in Fig. 3c together with calculated spectra for modelled structures with similar stacking characteristics. The peak positions matched well after application of a 10 cm^−1^ downshift to the calculated spectra. The assumed broadening of the calculated 3C spectrum closely matches the experimentally observed broadening. The experimental samples displaying hexagonalities of 0.20 and 0.36, respectively, all show significantly increased broadening compared to the 3C spectrum, becoming more prominent for the samples with higher hexagonalities. Furthermore, the Popigai samples display a notably asymmetric envelope with a longer ‘tail’ extending to lower wavenumbers, where features from (*hh*) and (*ch*) stacking are expected to appear. This indicates that the broadening is not solely a result of the small (5–30 nm) grain size, dislocations or strain, but can be attributed to peaks resulting from multiple different types of stacking disorder present within the sample, that cannot be resolved experimentally.

In general, our DFT results indicate that as soon as hexagonal stacking elements are introduced into the cubic diamond structure, the Raman spectra develop additional intensity at around 1300 cm^−1^, with weaker peaks observed down to approximately 1200 cm^−1^. The presence of a significant degree of cubic stacking is revealed by the maintenance of intensity in the 1330–1350 cm^−1^ region, although peaks for purely hexagonal stacking also occur within this range. Our conclusion is that although the appearance of Raman intensity in the 1200–1350 cm^−1^ range can give an indication that hexagonal layer stacking structures are present, it cannot be used as a primary diagnostic of the detailed structures that are present.

### HRTEM analysis

**Figure 4:**
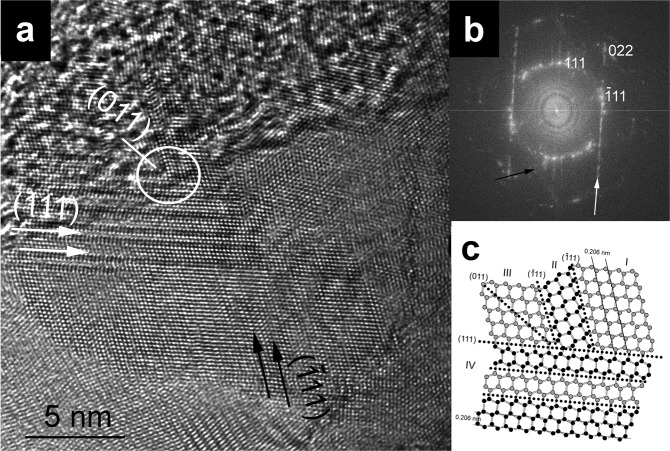
Abundant stacking faults and twins from a Popigai diamond (*Φ*_h_ = 0.20, *Φ*_cc_ = 0.79, *Φ*_hc_ = 0.85). (**a**) Multiple (111) and (−111) twins give rise to (011) intergrowth. (111) and (−111) stacking faults are marked with white and black arrows, respectively. A structural model of the white circled area is shown in (**c**). (**b**) Fast-Fourier transform calculated from (**a**) shows streaking of reflections (indicated by arrows) as a result of stacking disorder. (**c**) Idealized structural model of multiple (111) and (−111) twins. Twin modules (individuals) are represented by Roman numerals. The relationship between modules I and IV is the (011) mirror plane.

An atomic-scale interpretation of the structures present within diamond samples is only possible using HRTEM imaging combined with electron diffraction data analysis. We performed HRTEM analysis for the impact-produced Popigai diamonds. The samples studied consisted of ~5–30 nm-size aggregated grains consistent with previous findings^47^. The grains contained abundant 111 stacking faults and subnano-sized twins (Fig. 4), that gave rise to the complexity of stacking disordered diamond previously described for the Canyon Diablo sample^11^. Extensive defects divided the grains into nanosize domains and resulted in streaking of the 3C diamond reflections in the diffraction profiles. The HRTEM data confirmed the highly defective nature of the structures examined at the nanoscale, that gave rise to the globally averaged cubic/hexagonal stacking sequences deduced from the analysis of the X-ray diffraction and Raman spectroscopic data.

## Discussion

Our combined results lead to the following suggestions for a new protocol to be used for analysing and describing lonsdaleite, and various stacking disordered diamond materials recovered from meteor impact sites or produced in laboratory experiments. The first step of characterisation should be carried out using Raman spectroscopy. In absence of broadening of the main feature at ~1320 cm^−1^ accompanied by a tail towards lower wavenumbers, a diamond sample is unlikely to contain hexagonal stacking. However, analysing the spectra of materials that exhibit such features simply in terms of physical mixtures of 3C and 2H diamond phases or domains is too simplistic. Instead, the experimental spectra should be benchmarked against the library of calculated Raman spectra presented here across the stackogram (detailed information is presented in the Supporting Information). More robust structural insights can then be gained using powder X-ray diffraction. We suggest that the data should be obtained with high enough quality to then be examined using the MCDIFFaX approach, and the results plotted on a stackogram to ascertain the average hexagonality and the degree of clustering of cubic *vs* hexagonally stacked layer sequences. This procedure can then readily distinguish among samples that have experienced different degrees of shock, which implies different *P*-*T* conditions for the formation of the impact structure, or samples prepared in the laboratory^15,46^ (Fig. 2). Finally, we suggest that the final stage of the analysis should involve the use of HRTEM techniques to observe the actual nature of the layer stacking and the local structural defects that give rise to the nanoscale structures, that may not be visible to the X-ray diffraction or Raman spectroscopic examinations.

## Conclusions

We obtained X-ray diffraction data for a suite of diamond samples from the Popigai impact site, and analysed the results using the MCDIFFaX approach. This allowed us to plot the samples on a stackogram that indicated the overall degree of hexagonality and provided information on the clustering of cubic *vs* hexagonal layering within the sp^3^ bonded structures. DFT calculations of predicted Raman signatures for different cubic/hexagonal stacking sequences permitted a detailed interpretation of the different features occurring in the experimentally observed Raman spectra. Finally, HRTEM studies demonstrated the existence of multiple stacking faults along twin planes within the 3C cubic diamond structure giving rise to the overall observed hexagonal symmetry of selected Popigai samples. The results clearly indicate that these classic examples of lonsdaleite-containing impact diamonds are best described as materials containing intergrowths of cubic and hexagonally stacked layers at the nanoscale. Our suggested protocol for examining and cataloguing samples derived from previous and future studies will lead to a better standardised and more universally accepted description of what diamonds containing features indicating hexagonal stacking actually correspond to, and will aid in the geological interpretation of diamond-related materials recovered from impact sites.

## Methods

### Samples

A general description of the Popigai astrobleme, including its structure, geology, petrology of impactites, etc. is presented by Vishnevsky and Montanari^18^. The Popigai diamondiferous impactites (tagamites and suevites) are usually strong glass-bearing hard rocks made up of impact melt glass with various additions of target rock fragments, mainly Archean gneisses. The extraction of the impact diamonds was accomplished by crushing the rocks to a powder, heating in molten NaOH at 550 °C for 1 hour, then dissolving in aqueous HCl followed by washing with water. Most of the sample dissolves and the remainder consists of a small number of mineral grains, including the impact diamonds. These diamond grains were hand separated under optical microscopic examination. The Popigai impact diamonds are small (grain size usually 0.1 to 0.5 mm) and exhibit a range of colors from colorless, white, yellow, gray, dark-gray to black; yellow and dark grains are the most common (Fig. 1)^19^. Two main morphologies are observed: flattened and more bulky volume-xenomorphic grains. The carbon isotopic composition, δ^13^C_PDB_, ‰, of the Popigai diamonds was between −12.30 to −18.67, within the range of the target rock graphites. X-ray examination shows the Popigai diamonds are polycrystalline fine-grained aggregates showing evidence for preferred orientation. The crystallites vary between ~1 μm down to several nm in size. Although the cubic phase dominates, the diamonds exhibit varying degrees of hexagonality as described in this work.

### Experimental

X-ray diffraction experiments were carried out using a prototype instrument at the Department of Geosciences at the University of Padova^48^. The instrument consists of a Rigaku-Oxford Diffraction Supernova kappa-geometry goniometer equipped with an X-ray micro-source assembled with a Pilatus 200 K Dectris detector. The micro-X-ray source, MoK_α_ (*λ* = 0.71073 Å) operates at 50 kV and 0.8 mA (power = 40 W). Data collections were set up in powder mode, since the samples are polycrystalline aggregates, and the experiments were carried out in a phi scan mode over 360°. The diffraction data were fitted using the MCDIFFaX program (full details are given in the Supplementary Information)^37^. For measuring Raman spectra and recording HRTEM images, Popigai and cubic diamond samples were crushed using WC cubes. The Raman spectra were collected by directly measuring the diamond samples on the WC supports using a Renishaw inVia spectrometer with 325 nm laser excitation. For HRTEM analysis, a drop of isopropyl alcohol was placed onto the crushed diamond sample on the WC mount and a lacey carbon grid was swiped across to pick up sample. HRTEM imaging was conducted using a FEI Titan 80/300 STEM/TEM instrument equipped with a Cs (image) corrector and operated at 200 kV.

### Computational

Model structures with 48 layers and a range of different first-order stacking probabilities were prepared using our Stacky program^49^. First-principles calculations were then performed using the periodic DFT code CRYSTAL17^50^. Electronic exchange and correlation were described using the hybrid-exchange functional B3LYP. An all-electron atom-centred Gaussian basis set was used to describe the C atom, available from the CRYSTAL online database (www.crystal.unito.it), with the online label (C_6–21_G*_dovesi_1990). Reciprocal space was sampled using an 8 × 8 × 1 k-point mesh. The self-consistent field (SCF) procedure was performed up to a convergence threshold of ∆*E* = 10^−9^ Hartree (Ha) per unit cell. The Coulomb and exchange series were truncated with thresholds of 10^−7^, 10^−7^, 10^−7^, 10^−7^ and 10^−14^. Full geometry optimizations (lattice parameters and atomic positions) were performed using tight convergence criteria. The thresholds for the maximum and root mean square (rms) of the forces were set to 0.00015 Ha and 0.0001 Ha, respectively, and the maximum and rms of the displacements were set to 0.00045 Ha and 0.0003 Ha, respectively. Raman intensities were calculated using the coupled perturbed Kohn-Sham (CPKS) method^51^. The data are presented as orientationally and polarization averaged powder spectra.

## Supplementary information

Supporting Information

Supplementary Dataset 1

Supplementary Dataset 2
