# Supplementary material for: Quantifying hexagonal stacking in diamond
Source: Sci Rep. 2019 Jul 17;9:10334. doi: 10.1038/s41598-019-46556-3 (PMC6637244; doi:10.1038/s41598-019-46556-3)
Supplement: Supplementary file 1 — Supporting Information [file 41598_2019_46556_MOESM1_ESM.pdf]

Supporting Information

Quantifying hexagonal stacking in diamond

Mara Murri,[1] Rachael L. Smith,[2] Kit McColl,[2] Martin Hart,[2] Matteo Alvaro,[1] Adrian P. Jones,[3] Péter Németh,[4] Christoph G. Salzmann,[2*] Furio Corà,[2*] Maria C. Domeneghetti,[1] Fabrizio Nestola,[5] Nikolay V. Sobolev, [6] Sergey A. Vishnevsky, [6] Alla M. Logvinova, [6] Paul F. McMillan[2*]

[1]Department of Earth and Environmental Sciences, University of Pavia, Via A. Ferrata, 1 27100 Pavia, Italy; [2]Department of Chemistry, University College London, 20 Gordon Street, London WC1H 0AJ, UK; [3]Department of Earth Sciences, University College London, WC1E 6BT, UK; [4]Institute of Materials and Environmental Chemistry, Research Centre for Natural Sciences-HAS, Magyar tudósok körútja 2, 1117 Budapest, Hungary; [5]Department of Geosciences, University of Padova, Via G. Gradenigo 6, I-35131 Padova, Italy; [6]V.S. Sobolev Institute of Geology and Mineralogy, Siberian Branch of Russian Academy of Sciences Koptyug Ave., 3, Novosibirsk 90 630090, Russia

Contents

1 Preparation of the various diamond structures 2
2 Calculated Raman spectra 2
3 MCDIFFaX fitting of the X-ray patterns 2
4 Effect of hexagonality on the crystallographic *c*/*a* ratio 4
5 References 4

1 Preparation of the various diamond structures

Using our *Stacky* program,[1] a range of stacking disordered diamond structures were created with 48 layers. *Stacky* requires the target 1st order stacking probabilities as input and then produces a closest possible structure ensuring periodicity across the unit cell boundary in the *c* direction of stacking. As described in ref. 1 in detail, the requirement for periodicity across the boundary of the unit cell imposes some restrictions with respect to the possible stacking sequences.

In addition to the stacking disordered structures, the structures of the regular 2H, 3C and 4H polytypes were also produced with *Stacky*. The structural details of all created structures are listed in Table S1. The *structures.zip* file contains the *cif* files of the various structures.

Table S1. *Filenames, stacking probabilities and stacking sequences of the various diamond structures created with Stacky.*[1]

filename	Φ_c	Φ_{hc}	Φ_{cc}	stacking sequence (h = hexagonal, c = cubic)
diamond1.cif	0.25	0.25	0.25	hhhhhhhchhhcchhcchhchhchhhchhhhchhhhhhhchhhhcchh
diamond2.cif	0.5	0.5	0.5	hhccchhccchhcchhccchhhhchhchhchhchchccccchhchcch
diamond3.cif	0.75	0.75	0.75	cchcccchhccccchhhccccccchcchcchchccchcccccchcccc
diamond4.cif	0.5	0.25	0.75	hcccchhhhhhhhcchhcchhchcccccccchhhhhhhhhhccccccc
diamond5.cif	0.5	0.75	0.25	hhcchhcchchchcchchchcchhchhchchhcchchchchchchhcc
diamond6.cif	0.625	0.611	0.633	hhhcccchcchchcchchcchhccccchhchcccccccchccchchhh
diamond7.cif	0.875	0.833	0.881	cchcchcchcccccccccccccccccchcccccccccccccchhcccc
ccc.cif	1	1	1	ccc (cubic diamond)
hh.cif	0	0	0	hh (hexagonal diamond)
hchc.cif	0.5	1	0	hchc (4H polytype)

2 Calculated Raman spectra

The structures listed in Table S1 were used as starting structures for calculating the Raman spectra with CRYSTAL17 as shown in Figure 3 in the main article.[2] The calculated Raman spectra using Gaussian functions with 10 cm^{-1} half-widths as the profile functions are contained within *spectra.zip*.

3 MCDIFFaX fitting of the X-ray patterns

The recorded X-ray diffraction patterns were baseline corrected using shifted Chebyshev background functions within the GSAS software.[3] Great care was taken not to subtract intensity in the angle ranges where stacking disorder leads to diffuse scattering. The diffraction data were then fitted with the MCDIFFaX software by refining up to 2nd order stacking probabilities, lattice parameters and peak-profile parameters (*u*,

v, w and Gaussian / Lorentzian ratio).[4] During a typical refinement, a starting value of 0.5 was used for the zero-order stacking probability, and the 1st and 2nd order stacking probabilities were successively introduced during the refinement once the fits had converged using the lower-order stacking probabilities. Regarding the Cagliotti terms, the refinements were started by optimising w alone, whereas u and v were introduced during the later stages of the refinements. A detailed description of the MCDIFFaX program as well as the equations needed for the calculations of the 1st order stacking probabilities from the 2nd order stacking probabilities as well as the cubicity or hexagonality from the 1st order stacking probabilities are given in ref. 4. The determined stacking probabilities from all analysed Popigai diamond samples are listed in Table S2.

Table S2. *Second and first-order stacking probabilities together with the resulting cubicities and hexagonalities of the various Popigai diamond samples.*

sample	Φ_{ccc}	Φ_{hcc}	Φ_{chc}	Φ_{hhc}	Φ_{hc}	Φ_{cc}	Φ_{c}	Φ_{h}
POP1	0.865	0.330	0.786	0.665	0.756	0.709	0.722	0.278
POP2	0.873	0.471	0.822	0.969	0.845	0.788	0.799	0.201
POP3	0.932	0.337	0.847	0.883	0.852	0.832	0.835	0.165
POP4	0.563	0.695	0.789	0.449	0.680	0.614	0.638	0.362
POP5	0.934	0.330	0.815	0.907	0.830	0.834	0.833	0.167
POP6	0.842	0.901	0.817	0.895	0.830	0.851	0.848	0.152
POP10	0.786	0.367	0.616	0.842	0.687	0.632	0.651	0.349
POP11	0.931	0.748	0.613	1.000	0.721	0.915	0.895	0.105
POP12	0.781	0.359	0.459	0.766	0.586	0.621	0.607	0.393
POP13	0.912	0.343	0.765	0.963	0.804	0.796	0.797	0.203
POP16	0.822	0.833	0.832	0.724	0.811	0.824	0.822	0.178
POP17	0.773	0.916	1.000	0.907	1.000	0.802	0.834	0.166
POP20	0.849	0.916	0.950	0.831	0.943	0.858	0.869	0.131
POP21	0.809	0.880	1.000	0.898	1.000	0.821	0.848	0.152
POP23	0.783	0.914	0.920	0.854	0.914	0.808	0.827	0.173
POP24	0.778	0.844	0.975	0.926	0.973	0.792	0.824	0.176
POP25	0.798	0.893	0.955	0.921	0.953	0.816	0.838	0.162
POP27	0.936	0.876	1.000	0.878	1.000	0.932	0.936	0.064
POP29	0.381	0.743	0.796	0.981	0.828	0.545	0.645	0.355
POP31	0.796	0.889	0.970	0.902	0.968	0.813	0.838	0.162
POP32	0.959	0.860	1.000	0.789	1.000	0.955	0.957	0.043
POP34	0.802	0.396	0.794	0.948	0.822	0.666	0.711	0.289
POP35	0.761	0.426	0.685	0.892	0.739	0.641	0.673	0.327
POP36	0.786	0.898	0.941	0.727	0.924	0.807	0.828	0.172

4 Effect of hexagonality on the crystallographic *c/a* ratio

Within DIFFaX, the structure of a stacking disordered material is described by defining the structure of a single layer and then implementing the geometric recipes for stacking these layers together with the corresponding stacking probabilities.[5] Since MCDIFFaX deals with individual layers, the *c* height of a layer needs to be multiplied by 2 before comparing the *c*/*a* ratio with the one of the ideal hexagonal close-packed structure for which $c/a = \sqrt{8/3}$. For fully cubic diamond, the *c*/*a* ratio is $\sqrt{8/3}$ by definition because of the cubic symmetry. Figure S1 shows the *c*/*a* ratios of the various Popigai diamond samples as determined with MCDIFFaX and plotted as a function of the hexagonality. Despite some considerable errors, it can be seen that the *c*/*a* ratio increases with increasing hexagonality. This implies that the repulsive interactions within the six-membered rings in the boat conformation lead to slight separations of the stacked layers. The red line in Figure S1 shows the best linear fit to the data and the corresponding equation is shown in the legend. It is noted that the deviations from the fitted line do not seem to be connected with the deviations from random stacking, *i.e.* differences in the 1st order memory effects within the stacking sequences as shown in the stackogram in Figure 2B in the main article.

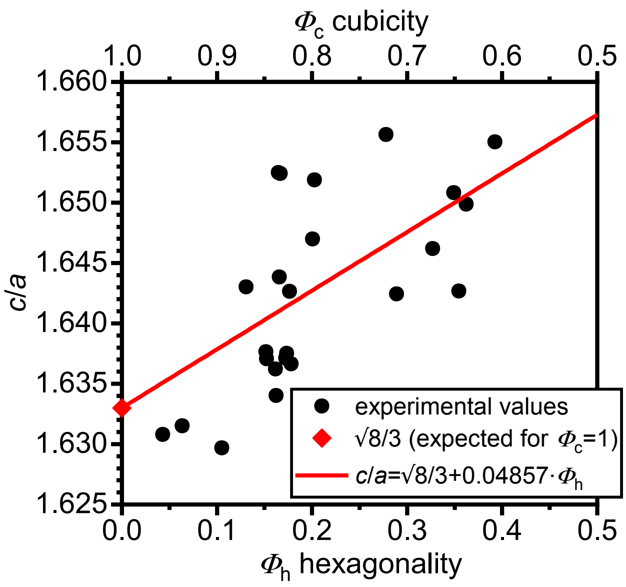

***Figure S1**. Crystallographic c/a ratios obtained from the MCDIFFaX fits of the Popigai diamonds. The red line represents the best linear fit to the data with the intercept value fixed to the expected c/a ratio of cubic diamond.*

5 References

1. Playford, H.Y., Whale, T.F., Murray, B., Tucker, M.G. & Salzmann, C.G.Analysis of stacking disorder in ice I using pair distribution functions. *J. Appl. Crystallogr.* **51**, 1211-1220 (2018).
2. Dovesi, R. *et al.* Quantum-mechanical condensed matter simulations with CRYSTAL. *Wiley Interdiscip. Rev. Comput. Mol. Sci.* **8**, 1-36 (2018).
3. Larsen, A. C.; Von Dreele, R. B. *GSAS - General Structure Analysis System*, University of California, (1985).

4. Salzmann, C.G., Murray, B.J. & Shephard J.J. Extent of stacking disorder in diamond. *Diam. Rel. Mater.* **59,** 69-72 (2015).

5. Treacy, M. M. J., Newsam, J. M. & Deem, M. W. A general recursion method for calculating diffracted intensities from crystals containing planar faults. *Proc. R. Soc.* **A 433**, 499-520 (1991).
